# Does Perception of Motor Competence Mediate Associations between Motor Competence and Physical Activity in Early Years Children?

**DOI:** 10.3390/sports7040077

**Published:** 2019-04-01

**Authors:** Charlotte J. S. Hall, Emma L. J. Eyre, Samuel W. Oxford, Michael J. Duncan

**Affiliations:** Centre for Sport, Exercise and Life Sciences, Coventry University, Coventry CV1 5FB, UK; hallc13@uni.coventry.ac.uk (C.J.S.H.); ab2223@coventry.ac.uk (E.L.J.E.); apx327@coventry.ac.uk (S.W.O.)

**Keywords:** physical activity, motor competence, preschool, children, young, perceived motor competence

## Abstract

Objectives: To examine if the relationship between physical activity (PA) and actual motor competence (MC) in British early years children is mediated by their perceived MC. Design: Cross-sectional convenience observational study. Methodology: MC was assessed with six locomotor skills (LC) and six object-control skills (OC) via the Test of Gross Motor Development-2. PA was measured via a wrist-worn triaxial accelerometer and PA grouped as daily total PA (TPA) and moderate-to-vigorous PA (MVPA). Perceived MC was assessed using the Pictorial Scale of Perceived Competence and Acceptance for Young Children. A total of 38 children (63% male; 37% female) aged between 3 and 6 years (5.41 ± 0.69) completed all assessments. Mediating impacts of perceived MC on the relationships between PA and MC were explored via backwards mediation regressions. Results: There were no mediating impacts of perceived MC on the relationship between PA and actual MC. Conclusions: The relationship between actual MC and PA is not mediated by perceived MC in a small sample of British early years childhood.

## 1. Introduction

Increased fatness in childhood is associated with obesity and obesity-related diseases in adulthood including cardiovascular disease, diabetes and some cancers, and is a public health priority to reduce [1]. Physical activity (PA) is one proposed solution to reduce overweightness and obesity [2]. Aside from reducing fatness, PA benefits such as reducing the risk of diabetes mellitus, cancer (colon and breast), and mental ill-health are well established [3]. The preschool (≤5 years) and early years (≤6 years) age is an opportune time for intervention as children in this age range are keen participators in PA, and new positive behaviors may be learnt to prevent unhealthy fatness [4]. Overweight children are less physically active than healthy weight children [5] and there is overall concern that children are not participating in enough PA for health benefits [6]. In order to promote effective PA engagement for health benefits, it is important to understand the correlates and mediators of PA [2,7].

A current conceptual model [5] proposes that motor competence (MC) is a precursor to PA and learning to move is necessary for PA participation and subsequent healthy weight. Actual MC, composed of locomotor MC (LC) and object-control MC (OC), is positively associated with PA engagement [2]. Children’s participation in PA influences MC, and in return MC influences PA [5]. Individuals develop MC in childhood to form the foundation for lifelong PA [8], specifically, learning to move effectively within space forms LC (running, galloping, skipping, hopping, sliding and leaping) and learning to manipulate objects forms OC (throwing, catching, bouncing, kicking, striking and rolling) [9]. Perceived MC is a proposed mediator of the relationship between actual MC and PA in early childhood [10,11]. There is a concern that children with lower perceived MC may lose motivations to participate in movement related tasks and reduce PA engagement [12]. However, whilst associations between PA and actual MC have been widely explored in young children [13], few have looked into the role that perceived MC plays on PA and actual MC. In children, perceived MC has been shown to be misaligned to actual MC, but as age increases, this misalignment declines [14,15]. Inaccuracies in perceived MC may be due to limited cognitive abilities that develop as children age. However, increased perceived MC is associated with increased PA [16,17,18] and children’s perceived MC should be considered when attempting to increase PA [18]. Perceived MC has correlations with PA [7,11] and with actual MC [2,4,14,19]. However, there is a paucity of studies investigating how self-perceptions of MC, collectively and as LC and OC, mediates associations between PA and MC, suggested by Stodden and colleagues [5].

One study tracked children from middle childhood (average age 10.1 years) over 6 years in Australian adolescents [17], to find that childhood MC and adolescent self-reported PA was mediated by perceived MC, suggesting the importance of perceived MC longitudinally. In a differing study [18] on Iranian females in middle childhood (8–9 years), it was found that perceived MC mediated the relationship between MC and self-reported PA. As did a further study [20] on 7–11-year-olds in Hong Kong, which found perceived MC mediated the relationship between LC and self-reported PA, but not with objectively measured PA. Nevertheless, one important study on Canadian preschoolers [21] highlighted that perceived MC does not mediate associations between actual OC and moderate to vigorous PA (MVPA), imperially confirming Stodden et al.’s [5] conceptual model in this cohort. Whilst this collective information provides some insight into the mediating potential of perceived MC on the relationships between PA and MC, no study to date as explored mediating effects in British early years children. 

Therefore, the aim of this study was to explore associations and mediating occurrences between PA, perceived and actual MC, and to evaluate perceived MC mediating effects between PA and actual MC in early aged British children (aged 3 to 6 years). The novelty of this study is to assess PA, actual and perceived MC in valid methods whilst separating components of PA and MC in a yet unresearched population. This study recognized that PA may be differentially associated with MC, so PA was explored as total PA (light, moderate and vigorous PA) and moderate-to-vigorous PA (MVPA), separately, according to current PA recommendations [1]. Additionally, this study recognized that LC and OC skills may be differentially associated within the theoretical model; therefore, MC was explored as total MC (all skills), LC skills and OC skills separately. This study used validated and objectively measured PA with cut-points validated in British preschoolers [22]. Additionally, an objective measure of actual MC was used [23] alongside a complimentary measurement of perceived MC [24].

## 2. Materials and Methods

### 2.1. Participants

Following institutional ethics approval, (P37708; 14 December 2015; Ethics Committee, Coventry University), an initial sample of 92 healthy early years participants from Coventry and Warwickshire were recruited. Preschools and schools were recruited using convenience sampling and were all state funded. Schools and preschools were recruited from varied socio-economic backgrounds, and all participants attended school or preschool for ≥15 h per week. Children were included in the analysis if all assessments were completed. Only 41% of participants had complete physical activity (PA) data, 94% had complete actual motor competence (MC) data, and 98% had complete perceived MC data. The final sample consisted of 38 children (4–6 years old; 5.37 ± 0.79).

### 2.2. Procedures

#### 2.2.1. Anthropometric Measures

Body mass (to the nearest 0.1 kg) and stature (to the nearest 0.1cm) were measured objectively by trained researchers using digital scales (SECA 875) (SECA Instruments, Ltd, Hamburg, Germany) and portable stadiometer (SECA 217) (SECA Instruments, Ltd, Hamburg, Germany), and gave body mass index (BMI: kg/m^2^).

#### 2.2.2. Motor Competence

Actual MC was assessed using the Test of Gross Motor Development-2 (TGMD-2) [23] in school facilities. Six locomotor (LC; run, jump, hop, leap, gallop and slide) and six object control (OC; catch, throw, kick, bounce, strike and roll) skills were assessed. Each skill comprised three to five components, and skill mastery on the TGMD-2 requires each component to be present. Video recordings of each skill (Sony Handicam CX405b, Sony, Weybridge, UK) were edited recordings into single-film clips of individual skills with Quintic Biomechanics analysis software v21 (Quintic Consultancy Ltd., Sutton Coldfield, UK). Each skill was described and demonstrated once by a researcher and each child performed each skill twice, following protocol for the administration of the TGMD-2 [23]. During analysis, each skill was marked by its individual components as successful (marked as 1) or unsuccessful (marked as 0) and totaled with both trials to give a total skill score. Scores were summed from two trials to create a total overall raw score (scored 0–96) following recommended TGMD-2 test administration guidelines [23]. The skills identified as LC and OC were grouped together according as subtest scores (LC scored 0–48; OC scored 0–48) and the summing of these gave a Total MC (TMC) score. All analyses were completed by two trained researchers. Intra- and inter-reliability was established for MC assessments within 15% of the final data set. Intra-rater reliability across MC, LC and OC showed 0.95, 0.93 and 0.81 agreement, respectively. Inter-rater reliability showed 0.71, 0.90 and 0.81 agreement across MC, LC and OC, respectively.

#### 2.2.3. Perceived Motor Competence

The pictorial scale of perceived movement skills competence for young children (PSMC) [24] based on the TGMD-2 [23] was used to assess perceived MC 24–48 h before MC was assessed. The PSMC uses a four-point Likert scale response variable (range 1–4). The PSMC is a reliable and valid measure of perceived MC in young children (aged 5 to 7 years) [24,25,26,27,28]. The children completed the perceived MC assessment one-on-one with a trained researcher with males and females receiving specific booklets [24]. For each skill, children were shown two, sex-specific, illustrations of a child performing the skill competently or incompetently and were then asked, “This child is pretty good at “skill”, this child is not so good at “skill”; which child is most like you?” Children were then asked to select a descriptive for further detail: 4—really good, 3—pretty good, 2—sort of good, or 1—not so good. Possible scores for perceived MC ranged from 12 to 48.

#### 2.2.4. Physical Activity

Physical activity (PA) was assessed over four consecutive days, including two weekend days, using wrist-worn triaxial accelerometery (GENEActiv, Activeinsights, Cambridge, UK) [29,30,31] on the child’s dominant wrist [31,32]. To meet the study inclusion criteria, children must have worn the accelerometer for ≥600 min during awake time, each of the four days recorded between 6 am and 10 pm; non-wear time was defined as ≥20 min of consecutive inactivity [33]. Children were shown how to use the accelerometers and the circumstances in which to remove them, such as during water-based activities and during sleep. The children were sent away with a leaflet to remind them of the correct procedures for wearing the accelerometers. Children wore the accelerometers on their dominant wrist which was identified by asking the children to write the first letter of their name. GENEActiv monitors have been validated for use in this population and has acceptable reliability [32,34].

Upon completion of the protocol, each participant’s accelerometer data was downloaded and stored on a computer. Using the GENEActiv post-processing software, the raw 100 Hz triaxial GENEActiv data were summed into a signal magnitude vector (gravity subtracted) expressed in 60 s epochs. The accelerometer counts were coded into sedentary (SPA < 8), light (LPA 8 < 9.3) and moderate-to-vigorous (MVPA ≥ 9.3) intensities using previously validated specific cut-points for the right wrist for preschool-aged children [22]. PA was then grouped into TPA and MVPA; this process was conducted to link the analysis in the present study to current recommendations for PA in early years children, to complete ≥180 min of TPA per day or complete ≥60 min of MVPA per day, or both. Current PA guidelines [1,35] recommend children under 4 years to complete ≥180 min of TPA and children aged 4–17 years to complete ≥60 min MVPA of per day.

### 2.3. Statistical Analysis

Descriptive statistics were calculated by all, sex groups, and weight-status, and were reported as means (± SD) (Table 1). Sex differences between all variables were assessed using independent t-tests. Analyses were conducted using IBM SPSS Statistics Version 21 (IBM Corporation, New York, NY, USA) with statistical significance set at *P* < 0.05.

To examine relationships between actual and perceived MC, LC and OC, and PA (TPA and MVPA), non-parametric mediation analyses were conducted [36]. Mediation regressions with either MVPA or PA as the outcome were performed with all predictor variables (MC and perceived MC). A series of mediation regressions with either actual or perceived MC as the outcome were then performed with all predictor variables (PA and MC or perceived MC). The significant influence of the mediating mechanisms (perceived MC, MC, and PA) were established where confidence intervals upper and lower bounds did not pass through zero [36]. Non-parametric mediation analysis using the Preacher and Hayes [36] model enables the assessment of direct and indirect relationships between two variables that may be mediated by an external variable. Using G*Power was required for parametric mediation, with *P* at 0.05, power at 80%, and to detect a large effect a total sample of 31 participants.

When presented in figures, each figure is bi-directional, so each PA (TPA or MVPA) or MC (total, LC or OC) variable can be the independent (X) or outcome (Y) variable. Depending on which mediation analysis is being presented, it may be either underlined or not. Solid lines represent the direct associations between two variables and dashed lines represent the mediating effects of perceived MC between those two variables.

## 3. Results

Table 1 reports the demographic information for all children, males and females. A total of 41% (n = 38) of the original 92 children completed all physical activity (PA), actual and perceived motor competence (MC) assessments and were therefore included in final analysis. Of this sample, 19% were overweight and obese.

There were no significant sex differences in MC (*P* = 0.08), LC (*P* = 0.95), MVPA (*P* = 0.72), TPA (*P* = 0.50) and perceived MC (*P* = 0.18). However, males were significantly more competent in OC skills (P = 0.03) than females and obtained themselves with significantly higher perceived MC (*P* = 0.04). However, weight status had no significant influence on MC (*P* = 0.26), LC (*P* = 0.20), OC (*P* = 0.56), MVPA (*P* = 0.31), TPA (*P* = 0.30) and perceived MC (*P* = 0.28). 

**Figure 1:**
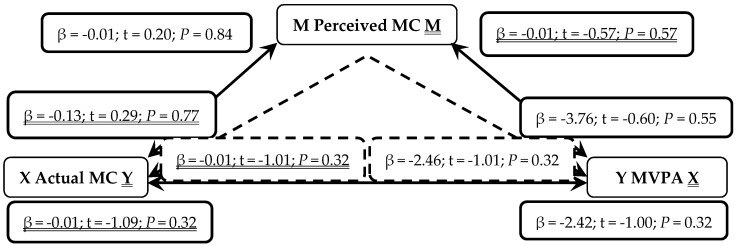
Mediation Analysis: Associations between actual motor competence (MC; Independent X and Outcome Y variable), perceived motor competence (MC, Mediator M) and moderate-to-vigorous physical activity (MVPA; Outcome Y and Independent X variable). Solid lines represent direct associations between mechanisms and dashed lines demonstrate indirect associations mediated through perceived MC.

Non-parametric mediation analysis, shown in Figure 1, found actual MC not to be a predictor of perceived MC (*P* = 0.84). Perceived MC was not a predictor of MVPA (*P* = 0.55). Actual MC was not a significant predictor of MVPA (*P* = 0.32). When controlling for the mediator perceived MC, actual MC was not a significant predictor of MVPA (*P* = 0.32). When the predictors were reversed, perceived MC was not a significant predictor of actual MC (*P* = 0.77). MVPA was not a significant predictor of perceived MC (*P* = 0.57). MVPA was not a significant predictor of actual MC (*P* = 0.32), even when mediated through perceived MC (*P* = 0.32).

**Figure 2:**
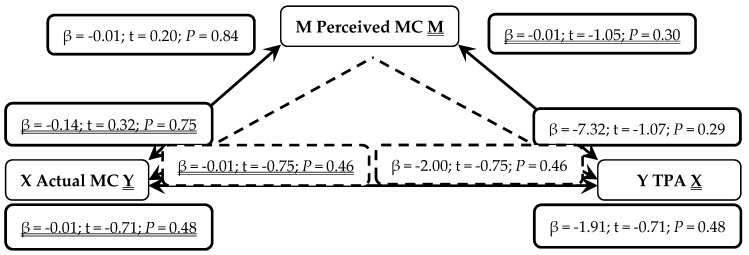
Mediated Analysis: Associations between actual motor competence (MC; Independent X and Outcome Y variable), perceived motor competence (MC, Mediator M) and total physical activity (TPA; Outcome Y and Independent X variable). Solid lines represent direct associations between mechanisms and dashed lines demonstrate indirect associations mediated through perceived MC.

Further analysis, shown in Figure 2, found perceived MC was not a predictor of TPA (*P* = 0.29) and neither was actual MC (*P* = 0.48). When mediating the relationship through perceived MC, actual MC was still not a significant predictor of TPA (*P* = 0.46). Therefore, in early years children, neither MVPA or TPA is positively associated with increased actual MC as mediated by perceived MC.

**Figure 3:**
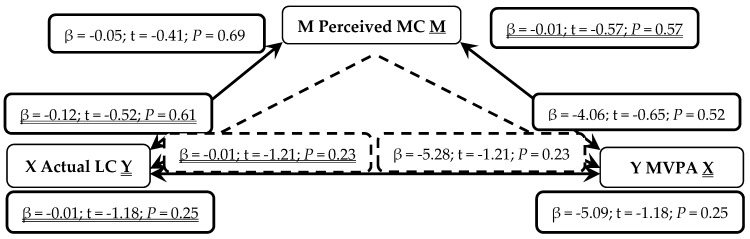
Mediated Analysis: Associations between actual locomotor motor competence (LC; Independent X and Outcome Y variable), perceived motor competence (MC, Mediator M) and moderate-to-vigorous physical activity (MVPA; Outcome Y and Independent X variable). Solid lines represent direct associations between mechanisms and dashed lines demonstrate indirect associations mediated through perceived MC.

In a second series of non-parametric mediation analyses, in Figure 3, actual LC was not a predictor of perceived MC (*P* = 0.69). Actual LC was not a predictor of MVPA (*P* = 0.25), even when mediated through perceived MC (*P* = 0.23). When reversed, perceived MC was not a predictor of actual LC (*P* = 0.61). MVPA was not a predictor of actual LC (*P* = 0.25) even when mediated through perceived MC (*P* = 0.23).

**Figure 4:**
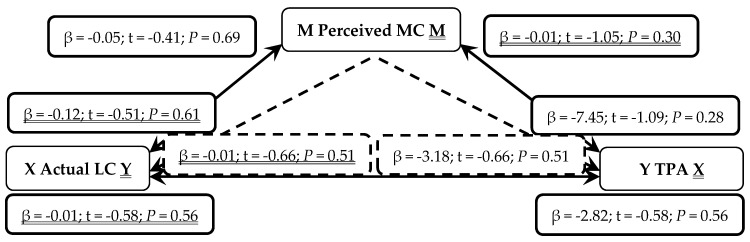
Mediated Analysis: Associations between actual locomotor motor competence (LC; Independent X and Outcome Y variable), perceived motor competence (MC, Mediator M) and total physical activity (TPA; Outcome Y and Independent X variable). Solid lines represent direct associations between mechanisms and dashed lines demonstrate indirect associations mediated through perceived MC.

Actual LC was not a predictor of TPA (*P* = 0.56) even when mediated through perceived MC (*P* = 0.51), as seen in Figure 4. When reversed, TPA was not a predictor of actual LC (*P* = 0.56) even when mediated through perceived MC (*P* = 0.51). Therefore, in early years children, neither MVPA or TPA is positively associated with increased actual LC as mediated by perceived MC.

**Figure 5:**
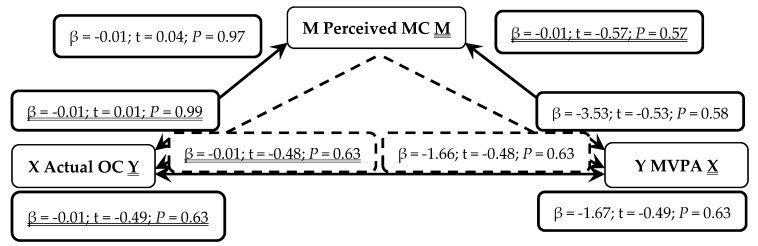
Mediated Analysis: Associations between actual object-control motor competence (OC; Independent X and Outcome Y variable), perceived motor competence (MC; Mediator M) and moderate-to-vigorous physical activity (MVPA; Outcome Y and Independent X variable). Solid lines represent direct associations between mechanisms and dashed lines demonstrate indirect associations mediated through perceived MC.

In a second series of non-parametric mediation analyses, in Figure 5, actual OC was not a predictor of perceived MC (*P* = 0.97). Actual OC was not a predictor of MVPA (*P* = 0.58), even when mediated through perceived MC (*P* = 0.63). When reversed, perceived MC was not a predictor of actual OC (*P* = 0.99). MVPA was not a predictor of actual OC (*P* = 0.63) even when mediated through perceived MC (*P* = 0.63).

**Figure 6:**
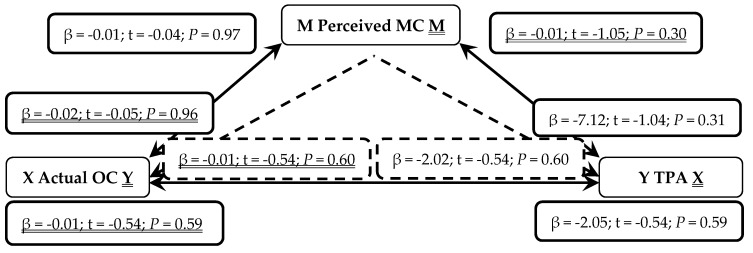
Mediated Analysis: Associations between actual object-control motor competence (OC; Independent X and Outcome Y variable), perceived motor competence (MC; Mediator M) and total physical activity (TPA; Outcome Y and Independent X variable). Solid lines represent direct associations between mechanisms and dashed lines demonstrate indirect associations mediated through perceived MC.

Seen in Figure 6, actual OC was not a predictor of TPA (*P* = 0.59) even when mediated through perceived MC (*P* = 0.60). When reversed, TPA was not a predictor of actual OC (*P* = 0.59) even when mediated through perceived MC (*P* = 0.60). Therefore, in early years children, neither MVPA or TPA is positively associated with increased actual OC as mediated by perceived MC.

## 4. Discussion

This study examined relations between physical activity (PA), and actual and perceived motor competence (MC) in early years British children, using objectively measured PA, validated and related measures of actual and perceived MC. No study to date has explored this topic in a British population. Stodden’s et al. [5] conceptual model suggests that early years children have limited accuracy of perceived MC, and generally show inflated perceived MC compared to actual MC [37]. It is expected that children under 7 years old would have limited cognitive ability to accurately distinguish between actual MC skill ability and effort [37].

In accordance with previous literature, there were significant sex-differences in perceived MC between males and females [38,39,40,41] as males’ perceived MC was significantly higher than that of females. Prior studies have tended to report that males perceive themselves more proficient in MC than females [38,39,40,41]. This current study suggests that this is the case in British early years children, and whilst not a novel finding in the literature, it has not previously been established in British early years children. If females have low perceived MC, then it is expected that PA will decline also [7,11,16]. Children in the early years are prior to puberty and therefore have limited biological differences between sexes; consequently, apparent differences between sexes must be from alternative factors, such as cultural and societal differences that may enhance or limit physical self-perceptions. Identifying that females have lower perceived MC than males, despite no significant sex-differences in MC, is important as inability to accurately predict perceived MC may impact on children’s PA and should be addressed when aiming to promote PA, highlighting early years females as potential target groups.

This study contributes new data to the area by empirically confirming Stodden’s et al. [5] conceptual model in British early years children. Perceived MC had no mediating effects on the relationships between PA (TPA or MVPA) and MC (total, LC or OC). Previous studies have reported mediating influences of perceived MC in children [18,20] but these findings were found in self-reported PA which is the perceptions of how active an individual is and is not a quantified result. Also, Khodaverdi’s et al. [18] and Chan’s et al. [20] investigations were conducted in older children. Older children that have transitioned from early to middle childhood, have developed cognitive capabilities for more accurate perceived MC [5]. Conversely, Crane, et al. [21] used accelerometery, the TGMD-2 and PSMC to assess MVPA, actual OC and perceived MC in Canadian preschoolers, to find that there were no mediating effects of perceived MC. Therefore, the mediating effects of perceived MC occur in middle to later childhood [18,20] not in early childhood [21].

However, this does not mean the role of perceived MC in early years children should be disregarded. Barnett et al. [17] found that perceived MC mediated the longitudinal associations between middle childhood PA and adolescent MC; additionally, actual and perceived MC are associated [2,4,12,19,42]. Therefore, inflated perceived MC may be an important component to encourage younger children’s MC engagement to influence future PA participation. Early years children may be more willing to persist in MC skills and PA, as at this age children struggle to differentiate between increased effort with increased performance, and will therefore engage in MC activities in which they believe they are skillful [43], which in turn increases PA engagement and such a link may not be seen via a cross-sectional study. It may be that current perceived MC leads to future PA and actual MC, as early years children are increasingly likely to participate in activities they perceived themselves to be good at [43]. According to Stodden et al. [5], the transition from early to middle childhood is an important developmental time when perceived MC grows more influential on actual MC and PA. Therefore, any mediating influence of perceived MC begins in later childhood beyond early years.

The use of TGMD-2 to assess MC in early years children is consistent with previous literature. Klingberg et al. [44] argues that the TGMD-2 is feasible in early years children. Cools and colleagues [45] compared MC assessment tools and concluded that the TGMD-2 was age-appropriate for this cohort. However, the cultural fit of the TGMD-2 in British children must be considered in this investigation. The TGMD-2 has many positives such as performance assessment and emphasis on OC skills, but Cools et al. [45] concludes that some skills may not be appropriate to use cross-culturally, such as striking, and the over arm throw are both related to baseball, which is not participated with as widely outside of the USA. Interestingly, even when some children were given explanation about the techniques to strike, many of them held the bat in a cricket stance rather than a baseball strike in accordance with TGMD-2, which impacted strike scores. When comparing between Flemish and American children [46], Flemish children scored significantly lower than Americans on the TGMD-2, concluding that cultural differences may explain this underachievement. Therefore, British children, who have limited exposure to baseball skills (strike) or basketball skills (dribble) and overexposure to soccer-based skills (kicking), may have limited exposure to all skills of the TGMD-2. 

Some methodological limitations need to be acknowledged. Firstly, the reduction from 92 to 38 participants with full data should be considered. Such a decline is not uncommon when dealing with early years children and, in the current study, was largely due to children not fully complying with the need to wear accelerometers for the minimum ≥600 min per day for four full days. This potentially highlights the practical challenges in collecting objective PA data in young children. However, this does mean that the findings of this study may have limited representability of the British early years population. Whilst the cohort were from varied socio-economic backgrounds, this sample may be too small to have generalized results for the early years age range. This may be due to inflated estimated effect size or have low reproducibility. Any findings from this study may have limited applicability and representation to generalize mediating effects in all British early years children. In the present study, at the time of analysis there was no method of sample size calculation for non-parametric mediation available, hence why the power calculation was based on that for a parametric mediation. This, however, should be acknowledged as a limitation as more recent literature [47] has suggested this approach will systematically underestimate the sample size needed to test the indirect effect and does not generalize to quantities from complex mediation models. Recognizing this, we subsequently determined the actual power of our sample using Monte Carlo Methods recently developed by Schoermann et al. [47]. A posteriori analysis indicated that with our sample of 31 participants actual power was 0.67. Future research examining this issue should therefore be aware of this and future work confirming the assertions made in this study with a larger sample are needed. Secondly, the cross-sectional nature meant data was recorded at one specific period and therefore causation cannot be established. Additionally, the accelerometers were removed for water-based activities and, therefore, may have underestimated PA; however, the accelerometers used have been identified as a valid method to capture PA.

The current study makes a novel contribution to current literature by confirming assertions made by Stodden et al.’s [5] conceptual model in a small sample of British early years children. Its strengths are the use of a sensitive process-orientated measure of MC (TGMD-2) [23] that is an appropriate method in this age group [44]. Additionally, PA was assessed objectively and using validated PA cut-points for preschoolers [22]. Furthermore, the PSMC was devised and based on the TGMD-2, and when used in conjunction provides uninfluenced comparison for actual and perceived MC. The PSMC has also been validated in early years children [24]. The consistent used of validated, objective measures in the present study allows for meaningful conclusions to be drawn. Finally, this study focused on 3-to-6-year-olds, which is a significant age group considering its closeness to adiposity rebound in children. This sample is relatively small; however, it is reflective of the difficulties in recruiting and completing objective assessments of PA and MC in early years children. In particular, capturing valid PA data of ≥600 min over multiple days is challenging in this population and other researchers should be mindful of potentially high attrition rates when assessing PA in young children (≤6 years old). Given that there was no mediating influence from perceived MC, the focus for early years education should be to encourage and develop children’s actual MC skills, before self-perception impacts children’s willingness to participate in movement-based activities. However, future work is needed to examine the temporal nature of any mediating effects of perceived MC on both actual MC and PA in a much larger cohort.

## 5. Conclusions

The aim of this study was to explore associations between PA, perceived and actual MC, and to evaluate perceived MC mediating effects between PA and actual MC in early aged British children (aged 3 to 6 years). This study empirically confirmed the assertions of Stodden et al.’s [5] conceptual model, where perceived MC had no mediating effects on actual MC (total, LC or OC) and PA (TPA or MVPA) in British early-years-aged children. These findings suggest that, in a small sample of children, the early years may be an important age range for intervention to encourage PA participation and development of actual MC, as it is before perceived MC has any influence on children’s inclination to participate in movement skills. It may be worth further investigation to address associations during longitudinal analysis to determine the long-term effects of each variable on current PA levels.

## Figures and Tables

**Table 1:** Descriptive values of BMI, Physical Activity, and Motor Competence.

Variables	All Children n = 38	Males n = 24	Females n = 14
Age (years)	5.37 ± 0.79	5.44 ± 0.68	5.36 ± 0.71
BMI (kg/m^2^)	16.48 ± 3.06	16.18 ± 2.53	16.28 ± 2.53
TPA (mins)	260 ± 148	274 ± 152	241 ± 152
MVPA (mins)	212 ± 126	219 ± 128	207 ± 128
TMC (out of 96)	69.88 ± 8.49	61.19 ± 8.56	58.01 ± 8.56
LC (out of 48)	33.32 ± 4.46	33.34 ± 4.89	33.28 ± 4.89
OC (out of 48)	26.57 ± 6.61 ^a^	27.85 ± 6.35	24.73 ± 6.35
Perceived MC (12–48)	39.24 ± 4.01 ^a^	39.81 ± 3.80	38.42 ± 3.80

^a^ Sex differences identified as significant at the 0.05 level (2-tailed). BMI, body mass index; TPA, total physical activity; MVPA, moderate-to-vigorous physical activity; TMC, total motor competence; LC, locomotor motor competence; OC, object-control motor competence; perceived MC, perceived motor competence.
